# Vasitis Nodosa: A Rare Diagnosis for Inguinal Swelling

**DOI:** 10.7759/cureus.13759

**Published:** 2021-03-08

**Authors:** Anum Sultan, Maria Hassan, Muhammad Saad Choudhry, Bhesham Shahani, Muhammad Ali

**Affiliations:** 1 Radiology, Dr. Ziauddin Hospital, Karachi, PAK; 2 General Surgery, Civil Hospital Karachi, Dow University of Health Sciences, Karachi, PAK

**Keywords:** male urogenital tract, vasitis nodosa, inguinal swelling, vas deferens

## Abstract

Vasitis nodosa involves benign, reactive spindle-shaped nodular thickening of the ductal epithelium of vas deferens. We report the case of a 21-year-old male with a history of bilateral undescended testes and left orchidopexy. The patient presented with the complaint of a non-tender left inguinal swelling. The definitive diagnosis of vasitis nodosa was made based on clinical evaluation and imaging findings. We suggest that this rare entity should be considered as a differential diagnosis of inguinal swelling during the assessment of the male urogenital system.

## Introduction

Vasitis nodosa refers to a benign condition, in which the ductular epithelium of the obstructed vas deferens undergoes reactive proliferation, forming fusiform nodular thickening. It may occur following an infection or either surgical or non-surgical trauma. Only 90 well-documented cases have been reported so far since the first description of the disease in 1943 [1,2]. Clinical evaluation of symptoms with detailed history, imaging findings, and follow-up are the key factors in the identification of vasitis nodosa and involves excluding differential diagnoses to prevent unnecessary surgery.

## Case presentation

Our patient was a 21-year-old male with no known comorbidities, who presented with the complaint of swelling in the left inguinal region for three months. He had a history of bilateral undescended testes for which he had undergone left orchidopexy at the age of eight years. Agenesis of the right testis was noted at the time of surgery. He had no history of fever, trauma, tuberculosis, alcohol consumption, cigarette smoking, or high-risk sexual behavior. On examination, there was swelling in the left inguinal region, which was non-tender on palpation. There was no associated erythema or pigmentation.

Laboratory investigations displayed serum electrolytes: sodium 142 mEq/L (normal: 136-149 mEq/L), potassium 4.6 mEq/L (normal: 3.8-5.2 mEq/L), chloride 104 mEq/L (normal: 98-107 mEq/L), and bicarbonate 24 mEq/L (normal: 23-29 mEq/L). The rest of the parameters were hemoglobin 14.2 g/dL (normal in males: 13.5-17.5 g/dL) and platelet count of 258,000/mcL (normal: 150,000-450,000/mcL). Prothrombin time was 11.5 seconds (normal: 10-13 seconds) and international normalized ratio was 1.05 (normal: 0.8-1.1). C-reactive protein was 12.4 mg/L (normal < 10 mg/L) indicating ongoing inflammation. His semen analysis revealed azoospermia (sperm number: normal >15 x 10^6^/mL) with a semen volume of 2.2 mL (normal >1.5 mL), seminal fructose of 15 µmol/ejaculate (normal >13 µmol/ejaculate) and pH of 7.4 (normal >7.2). His urine D/R showed a pH of 5.5 (normal: 4.5-8) with occasional leukocytes of 7 white blood cells (WBCs)/hpf (normal <2-5 WBCs/hpf).

**Figure 1:**
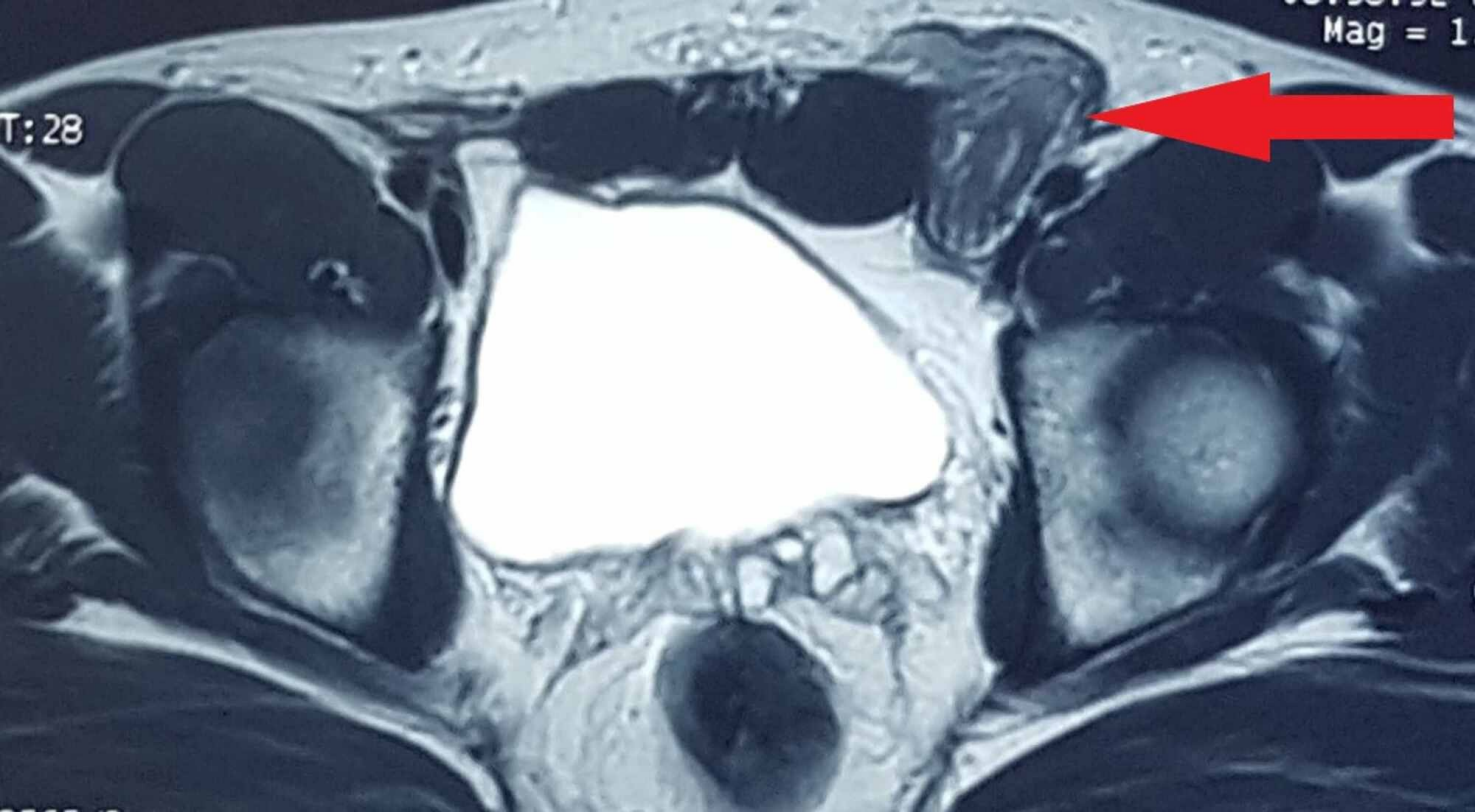
MRI T2-weighted images showing thickened left spermatic cord.

**Figure 2:**
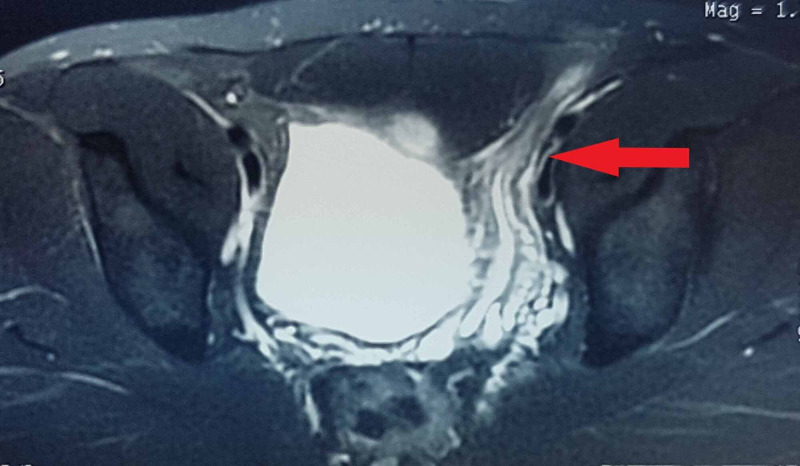
MRI T2-weighted fat saturation images showing a hyperintense tubular structure in pelvis representing thickened left spermatic cord.

**Figure 3:**
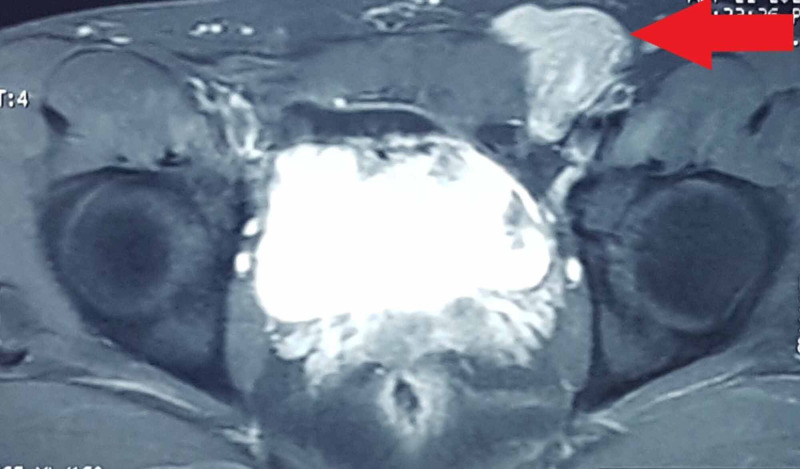
MRI T1-weighted post-contrast showing enhancement of left spermatic cord.

His initial MRI with contrast showed diffuse thickening of left vas deferens showing intense post-contrast enhancement. An initial diagnosis of vasitis was made, and antibiotic treatment was given. No response to treatment was noted [Figures 1, 2, 3].

**Figure 4:**
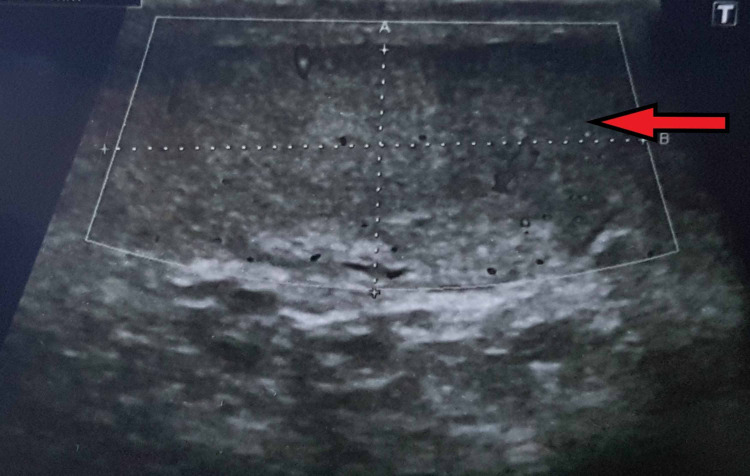
Ultrasound showing normal left testis.

**Figure 5:**
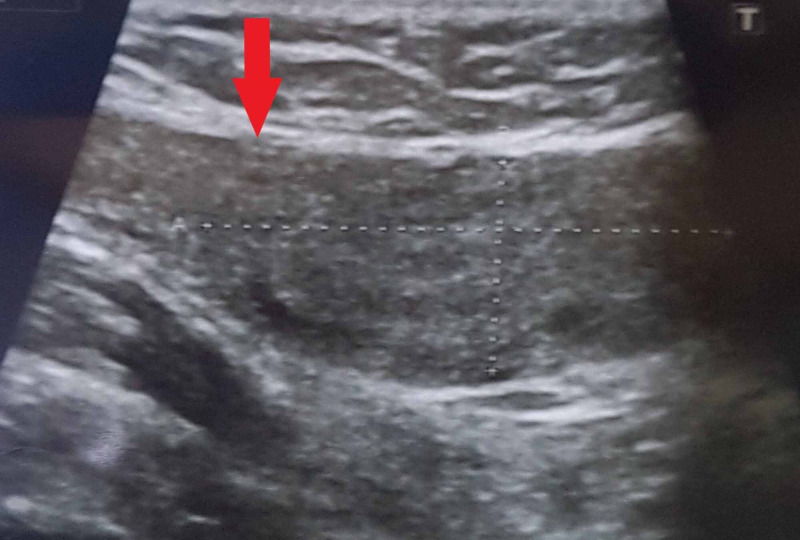
Hypoechogenic elongated nodular thickening of left vas deferens on ultrasound.

On three-month follow-up, an ultrasound was performed, which showed an elongated nodular hypoechoic area in the left inguinal canal measuring 5.0 x 1.3 cm showing central vascularity on color doppler imaging. Left testis and epididymis were normal. The right testis was not visualized due to agenesis [Figures 4, 5].

**Figure 6:**
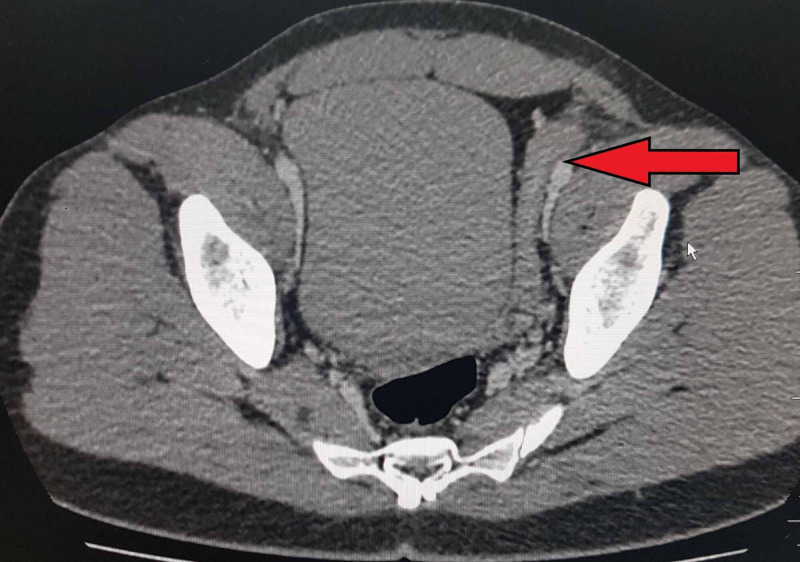
CT post-contrast showing enhanced left spermatic duct.

**Figure 7:**
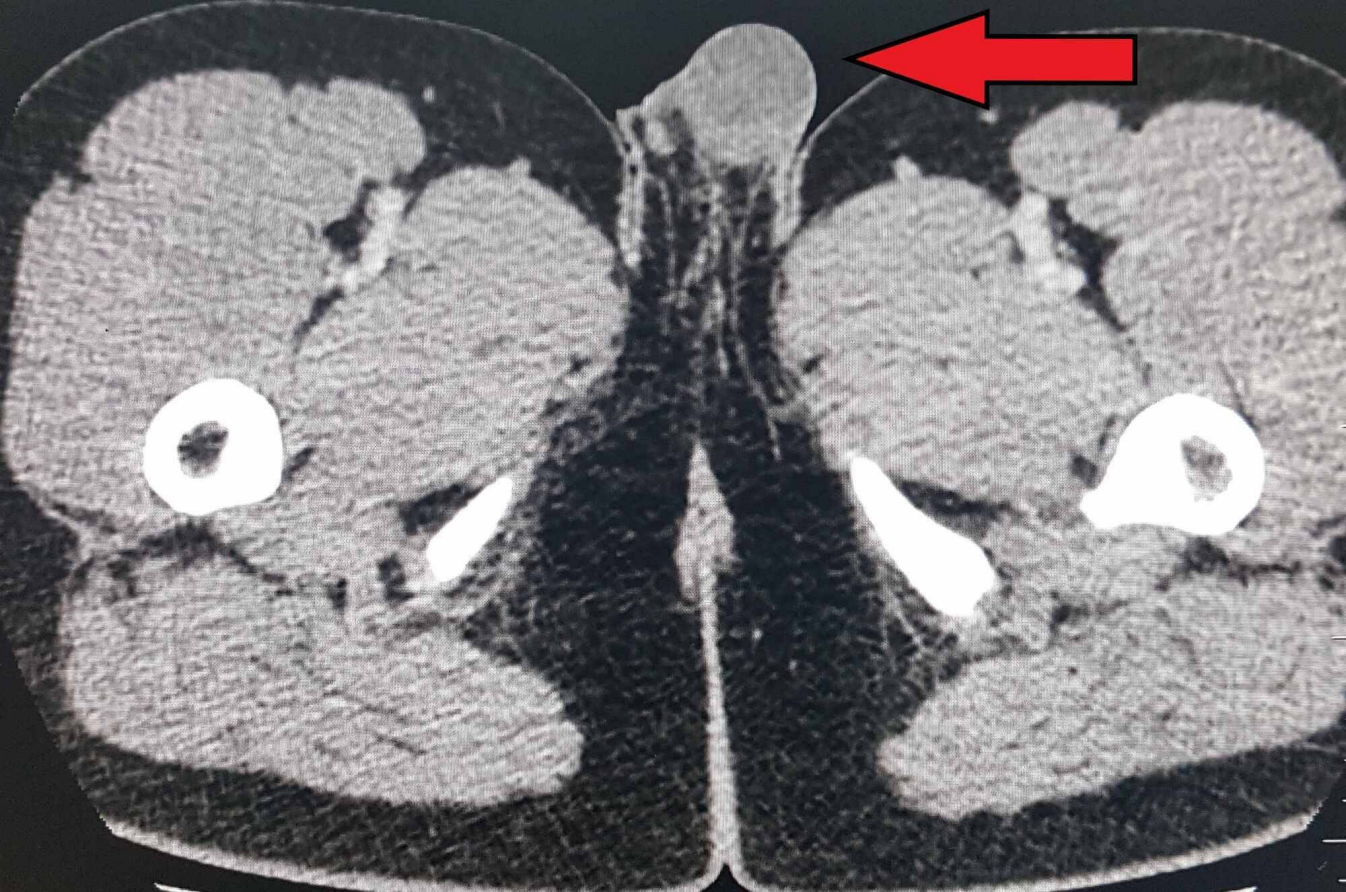
Left testis is visualized in the scrotum with agenesis of the right testis on CT.

His CT scan with contrast was arranged, which showed supra-scrotal nodular soft tissue thickening showing mild post-contrast enhancement of left vas deferens extending from the left seminal vesicle through the inguinal canal up to the scrotum. Widening of the left inguinal canal was also noted [Figures 6, 7]. Correlating the clinical symptoms, signs, and persistent imaging findings of the patient over three months, the diagnosis of chronic inflammation of vas deferens representing vasitis nodosa was made.

## Discussion

Vasitis is a rare entity characterized by Chen and Schlegel as acute painful infective vasitis and asymptomatic vasitis nodosa [1]. First described by Benjamin in 1943, vasitis nodosa is the benign chronic inflammation resulting in the fusiform nodular thickening of vas deferens [2]. The pathogenesis of the disease is believed to be secondary to an extensive proliferation of ductular epithelium with infiltration into the muscular layer and adventitia. This proliferation of epithelium results in luminal occlusion of vas deferens with increased intraluminal pressures causing leakage of spermatozoa and subsequent granuloma formation and fibrosis [3,4]. Most cases have a previous history of vasectomy or surgeries in close proximation of vas deferens, such as herniorrhaphy, perianal fistulectomy, or prostatectomy [5]. Vasitis nodosa has been reported in 66% of patients who had undergone vasectomy [1,6]. Our patient had undergone orchidopexy due to undescended testis, which was the most probable cause of vasitis nodosa in this case.

Clinical presentation is with asymptomatic inguinal swelling due to nodularity in vas deferens, which requires no treatment as seen in our patient. Various imaging modalities can be used for the evaluation of vasitis, which include ultrasonography, CT, and MRI. Ultrasonography is used to exclude common differentials, such as inguinal hernia, orchitis, epididymitis, and testicular torsion. On ultrasound, normal vas deferens is seen as non-compressible, anechoic, or hypoechoic tubular structure with total thickness ranging from 1.5 to 2.7 mm (mean, 1.89 mm) and a luminal diameter ranging from 0.2 to 0.7 mm (mean, 0.43 mm) [7]. Vasitis nodosa appears as hypoechoic thickened vas deferens, which may show minimal flow on ultrasonography. CT and MRI are more accurate in confirming the diagnosis and evaluation of the extent of vas deferens involvement, thus preventing extensive workup and unnecessary interventions required in other disorders affecting vas deferens and the male urogenital system.

 Kovi and Abgata have reported the benign neural invasion in cases of vasitis nodosa, emphasizing the fact that it can mimic low-grade adenocarcinoma of vas deferens [8]. The exact mechanism of neural invasion is not known; however, it is postulated to be due to entrapment of the ductal epithelium by the damaged regenerating nerve fibers after surgery [9]. Another possibility is the benign invasion of peripheral nerves by the proliferating ductal epithelium in an attempt for recanalization after vasectomy. Vasitis nodosa can be differentiated from adenocarcinoma on histopathology by identification of a preserved pattern of branching ductal proliferation with intraductal spermatozoa, identified on eosin and hematoxylin staining. Therefore, the pathologist should also be well aware of this rare entity to avoid misdiagnosis of vas deferens adenocarcinoma.

## Conclusions

Vasitis nodosa is a rare entity and should be considered as a differential diagnosis for inguinal swelling, especially in patients with a history of undescended testes, vas deferens, or perineal surgery. Clinical presentation with asymptomatic inguinal swelling resulting from vas deferens nodularity requires no treatment. CT and MRI scans can play a critical role in diagnosis to prevent unnecessary surgical procedures.
